# Redefining Mucosal Inflammation with Spatial Genomics

**DOI:** 10.1177/00220345231216114

**Published:** 2024-01-03

**Authors:** A.J. Caetano, P.T. Sharpe

**Affiliations:** 1 Centre for Oral Immunobiology and Regenerative Medicine, Barts Centre for Squamous Cancer, Barts and the London School of Medicine and Dentistry, Queen Mary University of London, UK; 2 Centre for Craniofacial and Regenerative Biology, Faculty of Dentistry, Oral and Craniofacial Sciences, King’s College London, London, UK

**Keywords:** oral mucosa, mucosal immunology, periodontal diseases, bioinformatics, multiomics, machine learning

## Abstract

The human oral mucosa contains one of the most complex cellular systems that are essential for normal physiology and defense against a wide variety of local pathogens. Evolving techniques and experimental systems have helped refine our understanding of this complex cellular network. Current single-cell RNA sequencing methods can resolve subtle differences between cell types and states, thus providing a great tool for studying the molecular and cellular repertoire of the oral mucosa in health and disease. However, it requires the dissociation of tissue samples, which means that the interrelationships between cells are lost. Spatial transcriptomic methods bypass tissue dissociation and retain this spatial information, thereby allowing gene expression to be assessed across thousands of cells within the context of tissue structural organization. Here, we discuss the contribution of spatial technologies in shaping our understanding of this complex system. We consider the impact on identifying disease cellular neighborhoods and how space defines cell state. We also discuss the limitations and future directions of spatial sequencing technologies with recent advances in machine learning. Finally, we offer a perspective on open questions about mucosal homeostasis that these technologies are well placed to address.

## Introduction

The function of all biological systems depends on the spatial organization of their cells. The inclusion of space and the notion that spatial fields of chemicals determine cellular specification dates to the 20th century (Wolpert 1969; Crick 1970). Physical position (i.e., the coordinates) of a cell within an organism and their environment ultimately determines cell identity, function, and physiological roles. During development, populations of cells interact and coordinate their behaviors in space and time to generate tissues and organs by integrating complex intracellular and extracellular cues, both chemical and mechanical, to make individual decisions on proliferation, differentiation, or migration (Gilmour et al. 2017). These individual decisions are ultimately the outcome of collective interactions; cells sense neighboring cells and their local environment to regulate various processes, such as cell cycle, cell shape, and gene expression (Snijder and Pelkmans 2011). It is thus essential to measure the various phenotypic and genetic states of single cells while also placing each cell in its environmental context to understand complex disease processes.

Abnormal tissue architecture or changes in cell organization and composition are hallmarks of disease and widely used as diagnostic tools. Understanding the relation between structure and function in tissues is ultimately essential to define how cellular organization reflects cells’ joint functionality. In this regard, barrier sites exposed to a wide range of environmental features that can influence plastic cell fate decisions, immune cell recruitment, and ability to respond to local environmental cues are of particular interest in decoding spatial connections.

To establish effective anatomical and functional barriers against commensal microorganisms and pathogens, mucosal surfaces rely on a highly integrated cellular organization, including structural and immunological systems that constantly monitor and respond to challenges. The oral mucosa constitutes a unique barrier site constantly exposed to low-grade chronic damage in the form of masticatory forces and a complex microbiome. The emergent technological advances that have redefined cellular identity, composition, and interactions promise a faster pace toward unravelling of the complex immune interactions at the core of mucosal physiology and disease. Until recently, high-throughput techniques could not be applied in situ, resulting in loss of spatial resolution. To address this, various creative methods were developed, such as microtomy sequencing (tomo-seq) (Junker et al. 2014; Wu et al. 2016), transcriptional profiling of physically interacting cells (Giladi et al. 2020), sequencing of cell clumps (Manco et al. 2021), or microdissection studies (Nichterwitz et al. 2016; Tirosh et al. 2016; Chen et al. 2017; Moor et al. 2018; Nichterwitz et al. 2018; Peng et al. 2019). Other approaches included machine learning methods to infer cells’ locations in whole-transcriptome single-cell RNA sequencing (scRNA-seq) data (Pettit et al. 2014; Achim et al. 2015; Satija et al. 2015). Together, these defined the need for whole-transcriptome spatially resolved methods, which led to the conception of spatial transcriptomics.

Here we review the principles of exploration of the data generated by these methods, examine the utility of spatial transcriptomics to study mucosal inflammation, discuss recent findings on human oral mucosa characterization, and highlight the promise of the technology for biological insights through integration with other modalities. We discuss relevant regional circuits in barrier tissues that typify an emerging paradigm of dynamic intercellular communication in the coordination of tissue function in space.

## Building Comprehensive Morphological Barrier Models

Deciphering tissue function and dysregulation in disease requires an upgrade from deconstructing tissues for their component parts—cells. In these models, tissue organization is described by the different variables that distinguish cells from each other. In dissociated single-cell profiling data, cellular phenotypes can be described based on overall gene expression, protein expression, or chromatin accessibility. While these data have overwhelmingly led to remarkable advances in certain disease areas, it is inherently biased (i.e., digestion protocols, cell loss) and fails to capture an essential feature of tissue biology, the cell microenvironment. This is a particular limitation when understanding, for example, immune cell function in disease; these are highly collaborative cells, and their phenotype is by large shaped by local signaling cues. For example, conversion of classical monocytes into nonclassical monocytes requires contact with endosteal vessels, and loss of structure and function of these vessels alters this conversion in aged mice (Bianchini et al. 2019).

Indeed, to understand cell phenotype, biologists have always relied on microscopy; Robert Hooke first defined cells in a cork sample using light microscopy in the 1600s (Hooke 1665). Functional spatial regulation in barrier tissues has been widely observed; for example, in the intestinal tract, cell function is tailored across different regions (James et al. 2020; Elmentaite et al. 2021). The palatal and intestinal stem cell compartments contribute to tissue self-renewal via distinct mechanisms depending on stem cell niche location (Ritsma et al. 2014; Byrd et al. 2019). Within the homeostatic lung, macrophage populations adapt and are shaped by the local environment, which allows them to fulfill the needs of their different anatomical niches (Mowat et al. 2017). Importantly, spatial determinants are not only chemical but also mechanical from changes in tissue architecture. For example, cells bordering a skin wound are exposed to altered substrate stiffness, which leads to reprogramming to a transitory progenitor-like state that restores the homeostatic function (Mascharak et al. 2021). In short, these studies suggest that different anatomical niches in the same tissue use distinct mechanisms to maintain their cell populations, and cell identity is not hardwired but determined by the coordination of intracellular and extracellular inputs from neighboring cells and the environment. To understand the functional importance of cell-to-cell variability within a tissue and to characterize processes that lead to cellular decision-making events, it is therefore important to account for spatial information when analyzing single-cell states.

**Figure 1.**
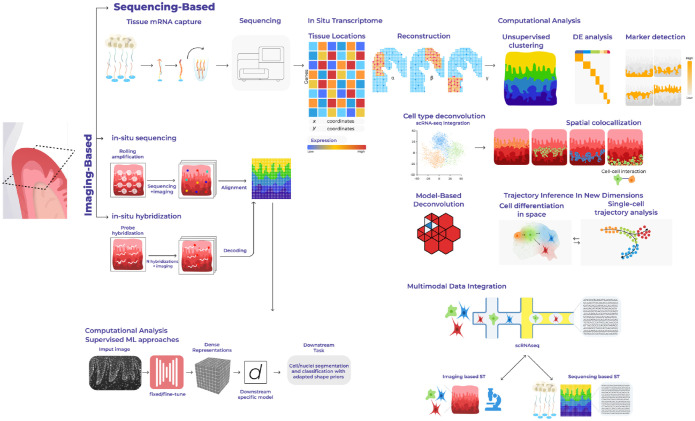
Overview of sequencing-based spatial technologies and computational analyses. Next-generation sequencing–based spatial transcriptomics methods use spatially barcoded messenger RNA–binding oligonucleotides. The product of spatial transcriptomics is the gene expression matrix, in which the rows and columns correspond to genes and locations. Computational or downstream analyses may include compositional analyses, cell-type mapping, colocalization studies, communication, and trajectory analyses in situ.

To conduct gene expression profiling of tissues at a comprehensive scale, sequencing-based spatially resolved transcriptomics (ST) methods propose solutions that involve capturing and quantifying messenger RNA (mRNA) population of molecules in situ (Fig. 1). There is a growing collection of available technologies with a wide spectrum of resolution and throughput (Table). These approaches can position mRNA molecules from aggregates of a few cells down to subcellular resolution, covering the transcriptomic landscape of tissue biopsies and even whole organs. Spatial transcriptome-wide profiling of healthy and diseased tissues allows for an unbiased discovery and hypothesis-generating research by not targeting specific genes or panel of probes. These observations—the presence of a cell type, a pattern of gene expression, or the colocalization of 2 cell states—may lead to a novel testable hypothesis, which should be validated independently.

**Table.** Spatial Transcriptomics Methods.

	Overview	Technique	Characteristics		Advantages	Disadvantages
Next-generation sequencing–based approaches	Spatially barcoded RNAs are collected and processed for sequencing. The barcode of each read is mapped to the spatial position, while the rest of the sequencing read is mapped to the genome to identify the transcript of origin, collectively generating a gene expression matrix.	Visium spatial gene expression	55-µm resolution translates to 3 to 30 cells per capture spot (first generation).		Whole-transcriptome capture—unbiased. Widely accessible.	Limited resolution (55-µm spot diameter with 100-µm center-to-center distance). Not single-cell resolution.
		Slide-seq, Slide-seq v2	Uses beads instead of capture spots, with the poly-T oligomers projecting radially around the bead; 10-µm diameter.		Whole-transcriptome capture—unbiased. High-resolution (10 µm) and sensitivity (500 transcripts per bead).	Not widely accessible.
		HDST (high-definition spatial transcriptomics)	2-µm diameter; direct improvement in resolution on spatial transcriptomics.		High resolution.	Not widely accessible.
		DBiT-seq	Deterministic barcoding, adopting microfluidics to apply poly-T barcodes to the tissue section.		Comapping of messenger RNA (mRNA) and proteins. 10-µm pixel size.	Not widely accessible.
		Stereo-seq	Uses randomly barcoded DNA nanoballs deposited in an array pattern to achieve nanoscale resolution.		Nanoscale resolution.	Not widely accessible.
		Seq-scope	Based on a solid-phase amplification of randomly barcoded single-molecule oligonucleotides.		Subcellular resolution spatial barcoding—it can be used to visualize nuclear and cytoplasmic transcripts.	Not widely accessible.
		PIXEL-seq	Polony (or DNA cluster)–indexed library sequencing.		High resolution.	Not widely accessible.
Imaging-based approaches	The image is processed to generate the gene expression matrix. To obtain a cell-level matrix, the image is segmented, either manually on small areas or systematically using a computational approach.	In situ sequencing–based methods.	Transcripts are amplified and sequenced in the tissue. 25 to 90 reads per cell, 69-gene panel.		In combination with whole-transcriptome approaches, provides high imaging resolution.	Targeted probes/biased approach. Genes of interest must be selected to design gene target-specific padlock probes.
		In situ hybridization (ISH)–based methods	A target sequence is detected by hybridization of a complementary fluorescent probe.	Multiplexed error-robust fluorescent ISH (MERFISH): 135-gene panel, ~100 reads per cell. Each gene has an associated binary code.	Allows investigation of intracellular organization and generation of large tissue maps. Able to detect around 10,000 genes at subcellular resolution.	Targeted/biased approach. Limited scalability.
				Sequential fluorescence ISH (seqFISH): 249-gene panel, ~30 reads per cell. Each gene has an associated color sequence code (24 color probes per gene) with 60 different pseudo-color options.	Imaging of mRNAs for 10,000 genes in single cells with high accuracy and subdiffraction limit resolution using a standard confocal microscope.	
				STARmap PLUS (spatially resolved transcript amplicon readout mapping): simultaneous RNA and protein localization.	10^6^ coding capacity to profile more than 20,000 genes.	

Tissue-wide processes can be computationally analyzed via spatial variance decompositions and other unsupervised techniques (Arnol et al. 2019; Argelaguet et al. 2020; Walter et al. 2023). Clustering approaches that account for spatial proximity (Zhao et al. 2021) or morphological similarity (Pham et al. 2020) could also be used to discover tissue models that go beyond molecular similarity. These clusters may correspond to distinct regions or cell types in the tissue of study, which can be annotated. Gene clustering can identify coexpressed gene modules corresponding to a cell type or cell state (Dries et al. 2021).

While capture resolution of next-generation sequencing (NGS)–based spatial transcriptomics continues to improve, widely accessible techniques remain low resolution (Visium spatial gene expression; Table), whereas high-resolution ones (slide-seq, high-definition spatial transcriptomics; Table) remain not as accessible to the wider research community. In addition, depth continues to be a limiting factor for spatial barcoding techniques. These limitations underscore the need to integrate current spatial transcriptomics platforms with other data modalities for better tissue characterization. For example, it is possible to use imaging-based techniques (Fig. 1; Table) that require gene probe panels to quantify transcripts of genes, such as multiplexed error-robust fluorescence in situ hybridization (MERFISH) or sequential fluorescence in situ hybridization (seqFISH), although novel transcripts not targeted cannot be detected. Alternatively, much attention has been given to the problem of inferring the cell-type composition of each spatial transcriptomics spot (deconvolution). Most methods achieve this by integrating single-cell data, generated either from the same sample (paired) or from a similar sample or database (unpaired) (Kleshchevnikov et al. 2022) and deconvolution of discrete cell types from spatial mixtures of NGS barcoding data (Zhao et al. 2021).

**Figure 2.**
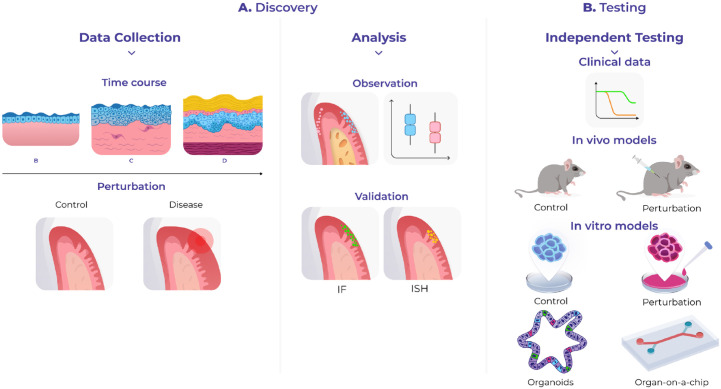
Study applications using spatial transcriptomics. Spatial transcriptomics can be used in discovery or hypothesis-driven science. (**A**) Examples of spatial transcriptomic data sets include normal tissue, a developmental or disease time course, and perturbation experiments. (**B**) Hypothesis testing applications. These can be further tested using clinical data or *in vivo or in vitro* models.

There are various downstream analyses that can be used, such as the generation of candidate cell–cell interactions that account for space; ligand–receptor pairs can be tested to understand which ones are more likely to be expressed by neighboring cells or spots or to find cells that colocalize (Arnol et al. 2019; Dries et al. 2021). Efforts have begun to develop systematized pipelines for spatial data processing, but more needs to be done to build standardized quality control measurements, data storage, and access pipelines to develop a common language in spatial transcriptomics. Likewise, extracting biological insights using computational tools requires greater collaboration between biologists and data scientists. This assumes particular importance when designing experimental methods to validate any generated hypothesis and gain a mechanistic understanding of cell function—for example, perturbation experiments using model organisms or complex culture systems able to mimic in vivo environments (Fig. 2).

Other aspects of cell function, such as the proteome and 3-dimensional (3D) chromatin conformation, have been developed to profile them along with the transcriptome of the same cells. To study the proteome, oligonucleotide-tagged antibodies can detect proteins of interest with single-molecule fluorescence in situ hybridization (smFISH)–based methods. For 3D chromatin conformation, MERFISH and seqFISH+ were developed to visualize chromatin structure by targeting DNA genomic loci or introns of nascent transcripts. Harmonization of these data with single-cell measurements through computational integration has given researchers an even more powerful tool to obtain comprehensive atlases of the cellular architecture of tissues (Fig. 1).

## Importance of Location and Tissue States in Disease

Disease exploits the responsive nature of immune and tissue-resident cells to their environment to drastically change the cellular organization of tissues. Highly inflammatory environments can increase the transition toward a specific disease-associated cellular state, and this transition can further influence the states of neighboring cells creating a feedback loop oriented toward pathological phenotypes. Therefore, spatial approaches that consider localization of a cell within a collective are important for understanding how tissue homeostasis is preserved and how disease is regulated.

Pathological cellular heterogeneity has been associated with distinct anatomical compartments determined by site-specific signaling cues. This influences the composition of populations in disease and their associated functions, which may explain the predisposition of anatomical sites to certain types of disease, for example, the tongue and buccal mucosa in oral squamous cell cancer or the junctional epithelium in periodontitis. Previously, we reported the colocalization of a proinflammatory fibroblast state in highly immunogenic regions adjacent to the sulcular and junctional epithelia (Caetano et al. 2023). These cells specifically express *CXCL10* and *CXCL8; CXCL10* is known to be significantly increased in periodontitis secreted in response to IFNγ and binds to its receptor *CXCR3*, modulating the recruitment of macrophages, T cells, natural killer (NK) cells, and dendritic cells. *CXCL8* is known to mediate and guide neutrophil recruitment and be a potent angiogenic factor (Heidemann et al. 2003). We hypothesize that the emergence of this fibroblast may act as a potential survival niche for immune cells, promoting local tissue destruction.

Transformation of tissue-resident fibroblasts by local malignant cues has been widely reported, and the relative composition of fibroblasts changes according to the anatomical and pathological environment. In the human oral mucosa, there is a selective loss and gain in periodontitis; we and others observed an heterogenous fibroblast composition across health and periodontitis (Caetano et al. 2021; Williams et al. 2021). With the development of image-based methods, we can now reconstruct cell state trajectories in space in response to disease, which promises new insights into understanding these pathological differentiation dynamics to understand disease progression.

Recent studies have also used spatial transcriptomics to describe the transcriptomic architecture of the intestinal mucosa in disease (Fawkner-Corbett et al. 2021; Parigi et al. 2022). Parigi et al. (2022) uncovered spatial molecular patterns that are present in health and that arise in response to damage, which coincided with different histological processes. This analysis identified distinct epithelial, lamina propria, and muscularis/submucosa genetic programs depending on their proximal to distal colon localization. Importantly, they also mapped human cells into the mouse ST data sets and observed conservation in transcriptomic features defining proximal and distal locations. Future studies addressing conservation between mouse and human oral mucosal molecular regionalization will be important.

Finally, the composition of barrier tissue microbiota is also known to be spatially distinct; oral microbes are increasingly viewed as specialized for individual habitats within the mouth, suggesting that the most significant factor that determines the niche for a microbe is its local habitat (Mark Welch et al. 2020), which in turn engages and shapes regional adaptive immune cell composition and function, highlighting the need to investigate oral mucosa cells in situ.

## Mapping the Human Oral Mucosa

The oral mucosa is one of the most complex barrier sites in the human body and serves a wide range of functions, including mastication, speech, and breathing, representing a major site of immune interactions. Tissue function requires the presence of various cell types and complex interactions between them. Breakdown in barrier mechanisms may lead to pathogen translocation and the development of chronic inflammatory disease, such as periodontal disease. Therefore, the oral barrier must quickly adapt to promote tissue regeneration and healing following injury. However, the cellular and molecular circuitry at steady-state conditions and how it adapts upon challenge only recently has been investigated. The recent development enabling genome-wide molecular profiling on a single-cell level has allowed us and others to generate detailed cellular maps of the human oral mucosa (Caetano et al. 2021; Huang et al. 2021; Williams et al. 2021), which provide invaluable open-access resources for the research and clinical communities (Caetano et al. 2022). However, detailed knowledge of mucosal anatomical physiology and cellular function *in situ* is a prerequisite to comprehensively investigate health and disease dynamics.

In our recent work, we overcome these limitations and integrate single-cell RNA sequencing data with spatial transcriptomics data sets of healthy and periodontitis-associated human samples. Spatial genomics data allowed us to uncover an unprecedented view of the molecular regionalization of the human oral mucosa, and by comparing tissue under steady state and periodontitis, we identified and spatially mapped transcriptional signatures of barrier homeostasis, immune cell activation/recruitment, and tissue remodeling. For example, within the junctional region, we consistently found a predominance of innate and adaptive immune cells compared to other anatomical regions. Moreover, targeted mapping of genes associated with periodontitis risk variants allowed us to infer their involvement in specific pathological processes based on their localization in areas with distinct histological properties (Caetano et al. 2023).

**Figure 3.**
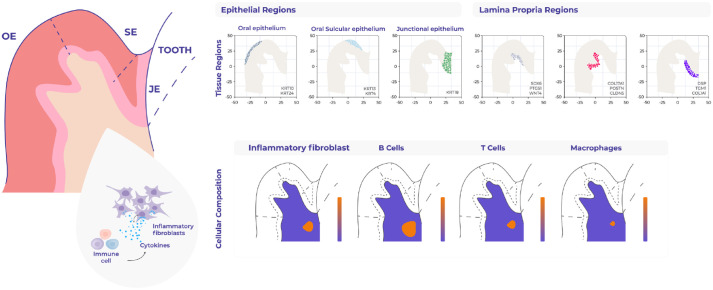
Mapping of human oral mucosa using spatial transcriptomics. Sequencing-based spatial technologies (10× Genomics) were used to characterize human oral mucosa in health and periodontitis (Caetano et al. 2023). Examples of this study include the unbiased gene expression mapping of the different epithelia and connective tissue regions. These data also allowed the mapping of individual cell types, such as the identification of a proinflammatory fibroblast, which colocalizes with several immune cells. This fibroblast state may contribute to disease progression by recruiting and maintaining lymphocyte populations.

The gingival epithelium consisting of the oral gingival epithelium (OGE), oral sulcular epithelium (OSE), and junctional epithelium (JE) also offers a unique opportunity to apply unbiased spatial techniques because its spatial organization is fundamental to its function. For example, the junctional epithelium (JE) provides a seal against oral microorganisms and expresses cytokines and chemokines, which are used not only for antipathogenic defense but also for maintaining physiological homeostasis. Our transcriptomic analyses identified these 3 distinct epithelial regions, which differ in not only keratin expression but also transcriptional regulators (Caetano et al. 2023) (Fig. 3). Furthermore, while the epithelial barrier provides structural defense, its function is largely influenced by the underlying connective tissue. Although initially considered a mere structural support, stromal cells, which include fibroblasts, endothelial/lymphatic cells, and neuronal and immune cells, are also actively involved in barrier maintenance through tissue remodeling, matrix deposition, neoangiogenesis, and production of proregenerative signals (Owens and Simmons 2013). Therefore, immune, epithelial, and stromal cells must quickly adapt within a defined microenvironment and establish a molecular network to promote tissue repair. We show that transcriptionally distinct stromal regions are associated with each epithelial region, suggesting a potential role in epithelial specification (Caetano et al. 2023). This finding has been supported by recent spatial proteomic analysis of periodontitis using multiplexed immunofluorescence (32-antibody) across healthy and periodontitis samples (Easter et al. 2023). This study suggests that the junctional region has higher innate cell concentration (neutrophils, macrophages, NK cells, dendritic cells), whereas the sulcular region shows different adaptive immune types (cytotoxic T cell, helper T cells, FOXP3^+^ regulatory T cells and B cells). Moreover, the subepithelial junctional stroma shows higher predicted interactions between macrophages and neutrophils. This work also highlights regional differences between gingival keratinocyte populations with associated distinct disease responses.

In addition to inflammatory processes, spatial technologies have also been applied to understand the tumor microenvironment in oral squamous cell cancer (Galeano Nino et al. 2022; Sun et al. 2023). One study, using a combination of 10× Visium spatial transcriptomics, single-cell RNA sequencing, and GeoMx digital spatial profiling, demonstrated how bacterial communities populate microniches that are less vascularized, highly immune-suppressive, and associated with malignant cells with lower levels of proliferation as compared to bacteria-negative tumor regions (Galeano Nino et al. 2022).

Overall, these initial studies already provided some clarity on the complex interplay between oral mucosa cell types throughout space and have potential implications for periodontal disease pathogenesis and for the engineering of in vitro systems.

## Conclusions and Future Perspective

Biology is defined by complexity—various components, nonobvious regulatory logic, stochastic interactions, dynamics over space, time, and physical stress. How to organize tissues into well-defined architectures or neighborhoods to understand functionality remains a goal to better understand and identify tissue distortion or disease. Spatial genomics tools have started to allow us to better compose tissue schematics to identify motifs or organizational neighborhoods that we can use to represent general assembly rules and create predictive models of disease initiation and progression.

Single-cell studies have provided a wealth of knowledge of the complex cellular landscape of the human oral mucosa (Caetano et al. 2021; Huang et al. 2021; Williams et al. 2021). They have detailed the spectrum of cell phenotypes and intercellular interactions in health and periodontitis. Spatial transcriptomics, while not yet at the single-cell with whole-transcriptome level, has already placed oral mucosa immune signatures in their tissue context and will be a key feature of future studies (Caetano et al. 2023).

To further address this complexity, recent technological advances have enabled the profiling of multiple biological modalities within individual cells. The growing list of modalities that can now be profiled at the single-cell level include proteome and metabolome (Stoeckius et al. 2017; Black et al. 2021; Rappez et al. 2021), transcriptome (Grun and van Oudenaarden 2015), and various aspects of the epigenome such as methylation (Karemaker and Vermeulen 2018), histone modifications (Ku et al. 2019; Bartosovic et al. 2021; Preissl et al. 2023), and chromatin accessibility (Lareau et al. 2019; Bartosovic et al. 2021). These technologies operate on dissociated cells, but progress has been made on the in situ assessment of the proteome (Giesen et al. 2014; Goltsev et al. 2018; He et al. 2022), epigenome (Deng et al. 2022; Lu et al. 2023), and joint profiling of the transcriptome and epigenome (Zhang et al. 2023) on histological tissue sections.

Computational integration of different data modalities remains a challenge, as well as common frameworks for the analysis of spatially resolved omics data. Recent pipelines were able to convert and harmonize data sets from multiple single-cell and spatial technologies in the developing human lower limb, creating a joint embedding of 3 modalities (Li et al. 2023), and others were able to bring the diversity of spatial data in a common data representation and provide a common set of analysis and interactive visualization tools (Palla et al. 2022). Comprehensive benchmarking studies to evaluate performance of each method provide valuable information and guidelines for the development of more effective methods. Furthermore, in standard ST data analysis, most models assume gene expression to have a constant variance across tissue locations; there is a need to model gene expression as a function of location or to assess if the noise of a gene’s expression is location dependent. A recent study proposed a new pipeline to model ST data using the Wilcoxon signed-rank test and false discovery rate (FDR) correction to identify genes with location-dependent noise variation in squamous cell carcinoma samples (Abrar et al. 2023).

Improved data collection to whole-mount and tissue-clearing protocols or more efficient computational protocols to align multiple sections should be developed to move from tissue sections to 3D analyses. The latter will depend on improved accessibility to more cost-efficient methods so they can be widely adopted and become an important and standard part of the field such as a hematoxylin and eosin staining.

Alternatively, advances in applied deep learning may generate new ways to address the challenge of routinely map single-cell profiles to histology. As an example, a model can be trained to generate a tissue’s bulk RNA sequencing profile from a histologic image (Schmauch et al. 2020), and more recently, single-cell expression profiles were inferred from histology from the same tissue sample by discovering a common latent space from both modalities across different samples (Comiter et al. 2023). In silico tissue data modality transfer between histological imaging and single-cell RNA sequencing domains would solve or increase accessibility of spatially resolved molecular profiles in terms of time, effort, but also expenses and to benefit from the insights provided by high-resolution methods in low-resource settings.

## Author Contributions

A.J. Caetano, contributed to conception, design, data acquisition, analysis, and interpretation, drafted the manuscript; P.T. Sharpe, contributed to conception, design, data acquisition, analysis, and interpretation, critically revised the manuscript. All authors gave final approval and agree to be accountable for all aspects of the work.
